# Acute Pleurisy in Horses1Read before the Illinois State Veterinary Medical Association, at Chicago, December 2d, 1902.

**Published:** 1903-05

**Authors:** A. H. Baker

**Affiliations:** Chicago, Ill.


### ACUTE PLEURISY IN HORSES.[1]

By Prof A. H. Baker,
CHICAGO, ILL.

Inflammation of the pleural membrane is very common in this climate. Most of the cases are sporadic, but a few of them are specific, being the localization of the specific fever of influenza. Sporadic pleurisy occurs as the original lesion and is uncomplicated in a majority of cases; but in many others it is seen in connection with pneumonia or rheumatic fever; then it exists oftentimes concomitantly with pericarditis. When uncomplicated it

[1] Read before the Illinois State Veterinary Medical Association, at Chicago, December 2d, 1902.

may take any degree of severity from a mild, circumscribed attack, being confined perhaps to a patch not more than two inches in diameter, to the involvement of the whole of the pleural surface. It may be right or left lateral or double. It may originate in either the costal or pulmonary pleura and extend to the other; but in most uncomplicated cases it probably arises in the costal, and the pulmonary becomes involved secondarily; but when occurring with pneumonia it probably arises in the pulmonary from extension to it from the parenchymatous tissue.

*Etiology.* In all probability there exists an inappreciable predisposing constitutional condition that directs the localization of the disturbance in the pleura; but the appreciable etiological conditions may be divided into idiopathic, traumatic, diathetic, and infective, and many cases occur deuteropathically.

The idiopathic cases arise usually from exposure to cold and dampness, especially when the temperature is suddenly lowered. In this connection it must be remembered that fatigue and exhaustion from hard work act as a predisposing cause, for a fresh horse, in most cases, would withstand the same exposure with impunity. The traumatic cases are due to direct injuries to the pleura by foreign bodies puncturing the chest wall; broken ribs lacerate or chafe it, and septic infection aggravates it. The diathetic causes are those which produce it by the localization of a predisposing constitutional condition, such as rheumatic fever and old age. The infective cases are those caused by specific disease, as influenza, irregular strangles. The deuteropathic cases include all of those that occur from extension of the inflammation from adjacent or contiguous tissues to the pleura, such as pleuro-pneumonia, in which the pneumonia is the original lesion and the pleurisy is secondary by extension.

*Special Pathology.* We divide the course of the disease into four stages, viz., first, congestion; second, dry inflammation or friction stage; third, stage of effusion, which we will divide into two parts; and, fourth, the stage of absorption. In the first stage the pleura becomes red in streaks or patches; these become confluent by extension in the course of an hour or two, when the pleuræ show a diffused redness. This stage runs rapidly into the second, when the pleuræ become dry by suspension of function and the friction sound is heard by auscultation. This stage is also short, being only about six hours in length, when it runs into the first part of the third, or exudative stage. At this time a plastic exudation occurs on the surface of the membrane, coagulates, and in some cases becomes adherent to its neighbor. In many cases the inflammation subsides at this time; the soft membrane softens through fatty degeneration, liquefies, and is absorbed; but if not, it runs into the second part, or stage of effusion, and large quantities of serum are poured out, more or less filling the pleural cavity, constituting what is known as hydrothorax. In cases that recover, the fourth stage follows and absorption of the effusion takes place slowly. The third stage is indefinite in length according to the severity and extent of the inflammation, but in a fair average case it is about eight or ten days. In this stage suppuration may take place and pus is mixed with the serum, known as empyema. All fatalities occur in this stage, either from asphyxia by the lungs being floated up to the back and interference with the action of the heart, or from general debility, prostration, and collapse from the absorption of the pus of the empyema. The fourth stage is long and tedious, requiring from four to eight weeks for absorption to take place to enable him to go to work.

*Symptoms.* It is often preceded by a rigor, and before it is fairly over sharp lancinating pains with rolling and sweating are manifested, resembling spasmodic colic; but if the pulse and temperature are taken they will be found to be accelerated, with a tinge of hardness and elevated to 104° or 105° F., which will distinguish it from colic, in which there are no disturbances of the pulse and temperature. As it runs into the second stage, the patient gets quiet, persistently stands with elbows turned out, abdominal muscles drawn tightly, producing the pleuritic line from the elbow along the cartilages of the ribs to the point of the hip. The breathing is careful and shallow and not labored, but is very painful, in which a grunt is emitted with nearly every expiration, and the grunt is particularly prominent if he is forced to move, especially if he is turned around shortly. A grunt in acute disease is always indicative of pleurisy. By auscultation a distinct friction sound is heard, and percussion causes pain, especially if the fingers are pushed into the intercostal spaces. The pulse runs about 60 and is small and hard; the temperature runs at about 105° F. The appetite is lost and the excretions are diminished in quantity and altered in character. The third stage is marked by more or less hydrothorax and dyspnœa in proportion to the amount of effusion. In a fatal case the nostrils are dilated, the flanks heave, the back is roached with each inspiration, the expired air is cold, the mucous membranes become livid, emaciation has been rapid and debility great ; he gets cold, sweat in patches more or less as death approaches. The diagnostic evidences of hydrothorax are dulness under percussion, absence of all sound by auscultation below the water line, and an increased respiratory sound above it. If the hydrothorax exists a week or longer the lower part of the chest becomes œdematous, especially between the forelegs, and the temperature persists at about 104° F.

*Treatment.* If seen in the first or second stage heroic treatment should be given, such as aconite, belladonna, spirits of nitrous ether, nitrate of potash, and acetanilid in liberal doses and repeated often for twenty-four to forty-eight hours, and apply smart counter-irritations over the sides of the chest, and repeat every twelve hours. After two days drop out the aconite and add nux vomica ; if it runs on to hydrothorax to a greater extent than one-third full of the chest, paracentesis thoracis is indicated. After the operation give iron and alcohol for two to four weeks, and nourishing diet. If empyema develops, as proven by the purulent character of the discharge from the canula at the time of the operation, rinse out the chest with a one-fifth of 1 per cent. solution of permanganate of potash to flush out the pus, then rinse again with a 1 per cent. aqueous solution of tincture of iodine.

